# Quantitative Classification of Rice (*Oryza sativa* L.) Root Length and Diameter Using Image Analysis

**DOI:** 10.1371/journal.pone.0169968

**Published:** 2017-01-19

**Authors:** Dongxiang Gu, Fengxian Zhen, David B. Hannaway, Yan Zhu, Leilei Liu, Weixing Cao, Liang Tang

**Affiliations:** 1 National Engineering and Technology Center for Information Agriculture, Jiangsu Key Laboratory for Information Agriculture, Jiangsu Collaborative Innovation Center for Modern Crop Production, Nanjing Agricultural University, Nanjing, Jiangsu, P.R. China; 2 Department of Crop & Soil Science, Oregon State University, Corvallis, Oregon, United States of America; University of Nottingham, UNITED KINGDOM

## Abstract

Quantitative study of root morphological characteristics of plants is helpful for understanding the relationships between their morphology and function. However, few studies and little detailed and accurate information of root characteristics were reported in fine-rooted plants like rice (*Oryza sativa* L.). The aims of this study were to quantitatively classify fine lateral roots (FLRs), thick lateral roots (TLRs), and nodal roots (NRs) and analyze their dynamics of mean diameter (MD), lengths and surface area percentage with growth stages in rice plant. Pot experiments were carried out during three years with three rice cultivars, three nitrogen (N) rates and three water regimes. In cultivar experiment, among the three cultivars, root length of ‘Yangdao 6’ was longest, while the MD of its FLR was the smallest, and the mean diameters for TLR and NR were the largest, the surface area percentage (SAP) of TLRs (SAP_T_) was the highest, indicating that Yangdao 6 has better nitrogen and water uptake ability. High N rate increased the length of different types of roots and increased the MD of lateral roots, decreased the SAP of FLRs (SAP_F_) and TLRs, but increased the SAP of NRs (SAP_N_). Moderate decrease of water supply increased root length and diameter, water stress increased the SAP_F_ and SAP_T_, but decreased SAP_N_. The quantitative results indicate that rice plant tends to increase lateral roots to get more surface area for nitrogen and water uptake when available assimilates are limiting under nitrogen and water stress environments.

## Introduction

Root systems serve an essential role in water and nutrient absorption [1–4]. Quantitative information about root morphological characteristics is useful for understanding the relationships between plant genetic expression and morphological plasticity and environments [5–9]. However, the soil environment prevents direct observation of roots, making sample acquisition and analysis of root morphology challenging and costly [10–12]. Limited information about roots is a serious constraint for understanding and describing the function of plant roots in water and nutrient uptake [7, 13, 14].

The common indices for describing root morphology are total dry weight, volume, surface area, and length. Measurement methods include displacement for root volume, staining for root surface area, line intersect for root length [15, 16], and microscopic observation for root diameter, especially for fine roots [17]. However, the information of root geometrical morphology obtained by these methods is not sufficiently detailed or accurate, especially for fine, fibrous root systems like those found in rice (*Oryza sativa* L.) and wheat (*Triticum aestivum* L.).

With the development of more advanced computer hardware and software, root analysis systems based on image analysis methods have become more widely used for washed root samples [18]. These systems make root morphology (such as diameter, length and surface area) measurement faster and more accurate compared with manual measurements. However appropriate parameters need to be determined and set in these systems and in root image analysis software like *WinRhizo* (Regent Instruments Inc., Québec, Canada) [19–21].

Root diameters of 0.5–2.0 mm are considered to be “fine” and those with diameters smaller than 0.5 mm are designated “very fine” [22]. These roots have high plasticity and potential to change their growth and development to adjust to a changing environment [23, 24]. Different root types have different functions [25, 26]. Large-diameter root usually is longer, less, and a main pathway to transports water and nutrient to shoot. By contrast, small-diameter root usually is shorter and more, and plays major role for taking up water and nutrient [27–29].

**Fig 1:**
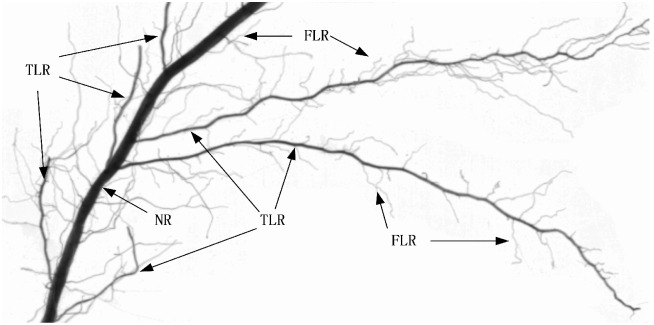
Image of rice root sample. TLR, FLR and NR denote: fine lateral roots, thick lateral root, and nodal root.

Rice is one of the world’s most important food crops [30, 31]. Most rice roots may be characterized as “very fine” (< 0.5 mm). Rice plants form fibrous root systems consists of an ephemeral seminal root, nodal roots, and their lateral roots. The seminal root originates from the embryo and lives only about 30 days through the early period of plant growth. Nodal roots emerge from the basal internode of stems and are called nodal roots (NRs). Lateral roots can be classified as fine lateral roots (FLRs) or thick lateral roots (TLRs). The TLRs may branch and form a new, higher order of lateral roots, while FLRs normally have no branches (Fig 1). The rice roots have no secondary thickening and the diameters were normally in the order of NR > TLR > FLR. NRs form the basic framework of the root system, with lateral roots providing a fine network [17, 32, 33]. The FLRs form the primary mechanism for absorbing water and nutrients, while TLRs help extend the entire root system for absorbing water and nutrients into a larger soil volume. Since different types of roots have specific functions, the ratios of NRs, FLRs and TLRs to total roots in response to phenology, soil water and nitrogen condition and crop management would be helpful for understanding plant root nutrition and water absorption [34].

Insufficient information about the dynamics of the length, diameter and surface area of different types of rice root under various management conditions was provided in the previous studies [32, 33]. Thus, based on the experiments involving different cultivars, N rates and water regimes in 2007–2009 by using image analysis software *WinRhizo*, the objectives of this study are to (1) study the dynamics of the length and diameter of NRs, FLRs and TLRs during growth period under cultivar, water and N treatments, and (2) determine the impacts of different water and N conditions on the surface area percentages of the three types of roots for helping understand rice root nutrition and water uptake.

## Materials and Methods

### Growing conditions and experimental design

Datasets from pot experiments were used to evaluate the effects of cultivars, N rates, and water regimes on root development. Experiments were conducted during 2007–2009 at the field experiment station of Nanjing Agricultural University, Nanjing, China (118°50′E, 32°02′N). Rice seeds were sown on May 20^th^ of each year, and thirty-day-old seedlings with a uniform size and vigor were transplanted into prepared pots. One plant was transplanted into each 60 cm high and 36 cm inside diameter PVC pot which contained a 2:1 mixture of air dried soil (*Hapli-Stagnic Anthrosol*) and sterilized sand. The air dried soil was sieved to pass through a 1 cm mesh. The mixture was soaked in water until it reached its water holding capacity. Pots were prepared one week before transplanting. The nutrient content of the soil mixtures for the various years is shown in Table 1.

**Table 1:** Organic matter, total N, P, and K content of soil mixtures used in 2007, 2008, and 2009 rice root experiments.

Year	Organic matter (g·kg^–1^)	Total N (g·kg^–1^)	Olsen-P (mg·kg^–1^)	Exchangeable K (mg·kg^–1^)
**2007**	12.8	0.86	10.2	105.2
**2008**	12.2	0.92	9.2	108.8
**2009**	13.6	1.12	11.1	112.4

Three cultivars including Nipponbare 14 (Japonica rice), Wuxiangjing 14 (Japonica rice), and Yangdao 6 (Indica rice) were evaluated in the cultivar experiments (Exp. 1 and 2), while Wuxiangjing 14 was evaluated in the nitrogen (Exp. 3 and 4) and water (Exp. 5 and 6) experiments.

N was provided as urea, split into 4 applications: 50% prior to transplanting, 20% at the onset of tillering, 20% at spikelet initiation, and 10% at the beginning of the grain filling period. Before transplanting, soil used for all treatments received a basal application of monocalcium phosphate and potassium chloride at rates of 80 kg P_2_O_5_ ha^−1^ (0.8 g per pot) and 150 kg K_2_O ha^−1^ (1.5 g per pot).

Three water regimes were established 15 days after transplanting as 1–2 cm water layer, wetting irrigation (−20 ± 5 kPa soil water potential), and dry cultivation (−40 ± 5 kPa soil water potential). Daily soil water was adjusted as needed after measurement of soil water potential using an EQ15 equitensiometer (Ecomatik Instruments, Germany).

The cultivar, N rate and water regime in each experiment were showed in Table 2.

**Table 2:** Experiments and treatments.

Experiment (Year)	Treatment	Cultivar	N rate (kg N ha^−1^)	water regime
**Exp.1(2007) & 2(2009)**	V1	Nipponbare	150	water layer
	V2	Wuxiangjing 14	150	water layer
	V3	Yangdao 6	150	water layer
**Exp.3(2007) & 4(2008)**	N1	Wuxiangjing 14	0	water layer
	N2	Wuxiangjing 14	150	water layer
	N3	Wuxiangjing 14	300	water layer
**Exp.5(2007) & 6(2008)**	W1	Wuxiangjing 14	150	water layer
	W2	Wuxiangjing 14	150	wetting irrigation
	W3	Wuxiangjing 14	150	dry cultivation

### Sampling

After transplanting, roots were sampled at 7 to 12 days’ intervals, and three plants were sampled for each treatment. Roots were washed by tilting pots and carefully spraying with water until the attached soil and sand particles were removed. A 0.5 mm diameter sieve was used to prevent loss of fine roots during washing. Samples were labeled, placed in plastic containers with lids, and stored in a 4°C refrigerator. If not analysed immediately, root samples were immersed in a 35% aqueous ethanol solution. For analysis, roots were excised from main stem and tillers, placed in a plexiglas trays (200 by 300 mm) with a 4 to 6 mm deep layer of water, and spread out with tweezers to minimize overlapping.

### Root image analysis

The root length and diameter were measured by software *WinRhizo* (version 5.0) software. Grayscale images (400 DPI) of roots were obtained using an EPSON1680 scanner. Three types of rice roots were evaluated in this study: nodal root (NR), fine lateral roots (FLR) or thick lateral root (TLR). Their mean diameter, length and surface area were obtained based on their diameter ranges which were determined by random sampling and statistical comparison method. The details of root image analysis method were described in S1 File. And the relevant raw data are available in S2, S3 and S4 Files.

### Statistics analysis

Data were subjected to a one-way ANOVA and significant differences of treatment means were compared by the Fisher's protected least significant difference (LSD) procedure with the SPSS 17.0 (SPSS Inc., 2008).

## Results

### Dynamics of mean diameter of different types of rice roots with growth stages

**Fig 2:**
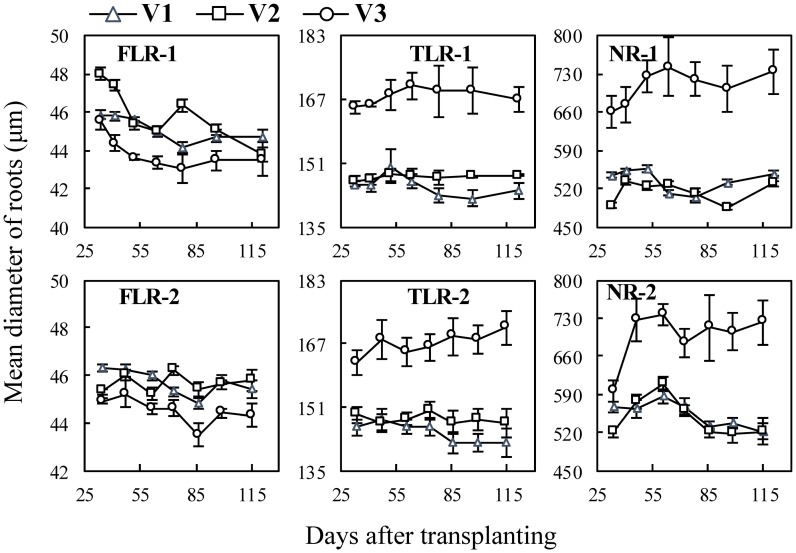
Changes in mean diameter of three types of rice roots in cultivar experiment following transplanting. FLR, TLR and NR denote different root types: fine lateral roots, thick lateral roots, and nodal roots, respectively. The numbers following the letters denote Exp.1 and 2. Cultivars treatments in Exp.1: V1, ‘Nipponbare’; V2, ‘Wuxiangjing 14’; V3, ‘Yangdao 6’. The values are the means of 3 replicates (± *SE*).

The dynamics in the mean diameter of three types of rice root at different growth stages were analysed using the data obtained by *WinRhizo*. In the cultivar experiment (Fig 2), the mean diameter of FLRs of V3 was smallest and those of TLRs and NRs were the largest at different growth stages. V1 and V2 did not differ significantly in root mean diameter for any of the three root types. For example, the mean root diameters of TLRs and NRs of V3 were in the 165.2–170.4 μm and 661.5–743.3 μm ranges, respectively, but for V1 and V2, values were 142.3–150.3 μm and 486.3–556.3 μm (Fig 2 TLR-1, NR-1, TLR-2, and NR-2).

**Fig 3:**
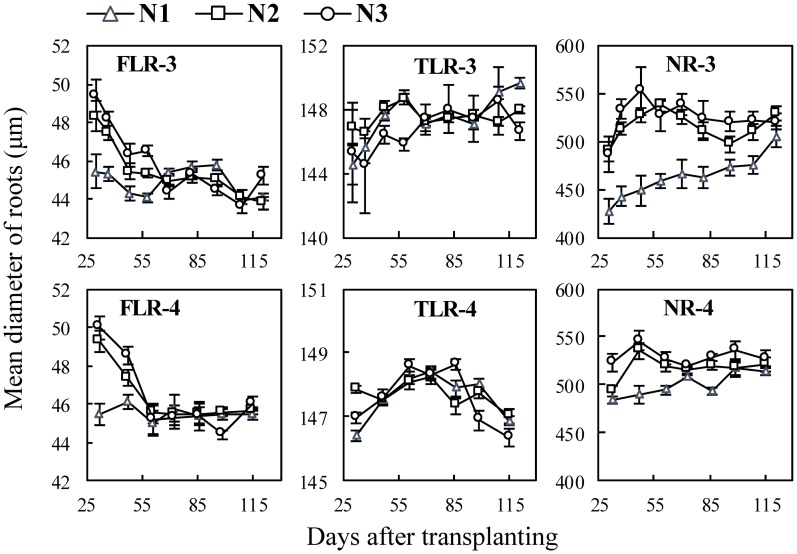
Changes in mean diameter of three types of rice roots in N experiments following transplanting. FLR, TLR and NR denote different root types: fine lateral roots, thick lateral roots, and nodal roots, respectively. The numbers following the letters denote Exp.3 and 4, and the cultivar ‘Wuxiangjing 14’ was used. N1, 0 N; N2, 150 kg N ha^−1^; N3, 300 kg N ha^−1^. The values are the means of 3 replicates (± *SE*).

In the N experiments (Fig 3), most of the differences of the mean diameters of the FLRs and NRs occurred between 0 N (N1) and applied N (N2 and N3), but no significant differences between N2 and N3 (Fig 3 FLR-3, NR-3, FLR-4 and NR-4). In Exp. 3, the mean diameters of NRs of N1 were 427.4–505.4 μm, and with applied N, the range was 486.9–552.9 μm (Fig 3 NR-3). The mean diameters of TLRs did not differ significantly among three N treatments (Fig 3 TLR-3 and TLR-4).

**Fig 4:**
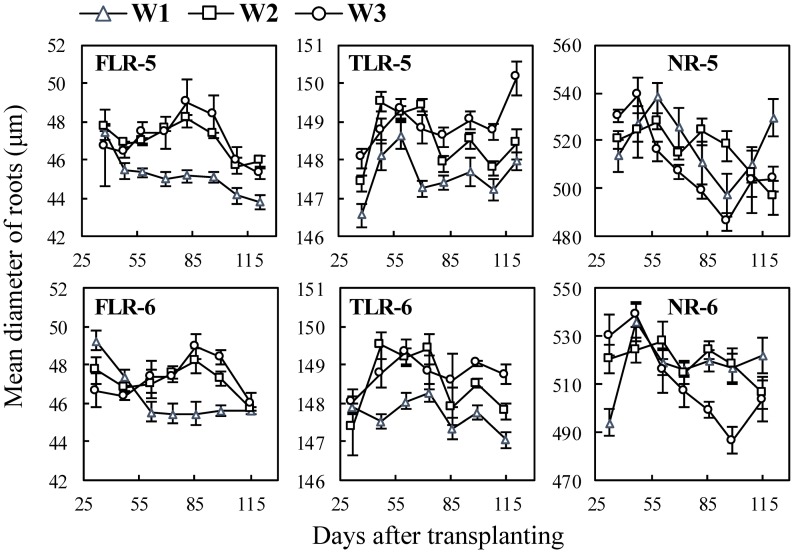
Changes in mean diameter of three types of rice roots in water experiments following transplanting. FLR, TLR and NR denote different root types: fine lateral roots, thick lateral roots, and nodal roots, respectively. The numbers following the letters denote Exp.5 and 6, and the cultivar ‘Wuxiangjing 14’ was used. W1, keeping 1–2 cm water layer; W2, wetting irrigation; W3, dry cultivation. The values are the means of 3 replicates (± *SE*).

In the water experiments (Fig 4), mean root diameters of FLRs and TLRs under W1 treatment were generally smaller than those under W2 and W3 (Fig 4 FLR-5, TLR-5, FLR-6 and TLR-6). For example, after 48 DAT, the mean diameters of FLRs under W1 were 43.8–45.4 μm but under W2 and W3, root diameters were 45.3–49.3 μm (Fig 4 FLR-5). And the mean diameter of NRs under W3 was larger than those under W1 and W2 at the early stage of the treatment.

### Dynamics of length of different types of rice roots with growth stages

**Fig 5:**
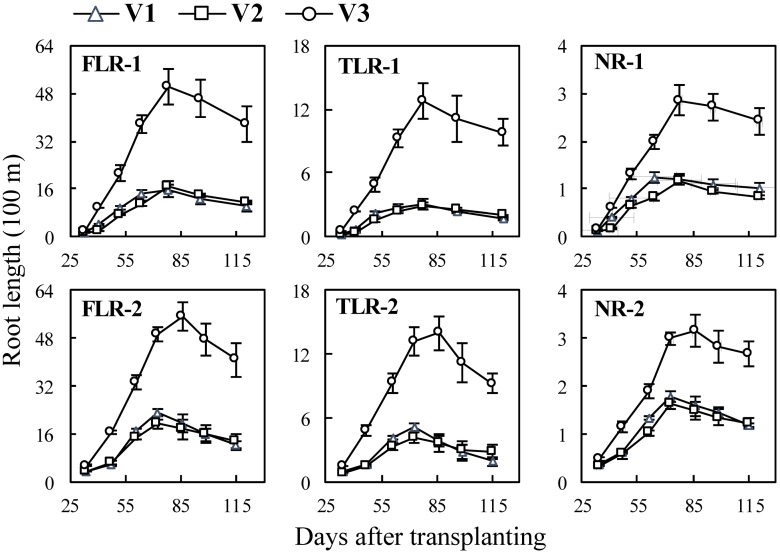
Changes in length of three types of rice roots in cultivar experiments following transplanting. FLR, TLR and NR denote: fine lateral roots, thick lateral root, and nodal root, and the numbers following the letters denote Exp. 1 and 2, respectively. V1, ‘Nipponbare’; V2, ‘Wuxiangjing 14’; V3, ‘Yangdao 6’. The values are the means of 3 replicates (± *SE*).

In the cultivar experiments (Fig 5), the length of three types of root increased rapidly and reached or approached their maximum values around heading stage, and then increased slowly or decreased. Root lengths of V3 increased faster significantly than those of V1 or V2 (*p*≤0.05). For example, at 78 days after transplanting (DAT) in Exp. 1, the length of FLRs of V3 was 3.11 times the mean value of those of V1 or V2 (Fig 5 FLR-1). Root lengths of V1 and V2 did not differ significantly (*p>*0.05).

**Fig 6:**
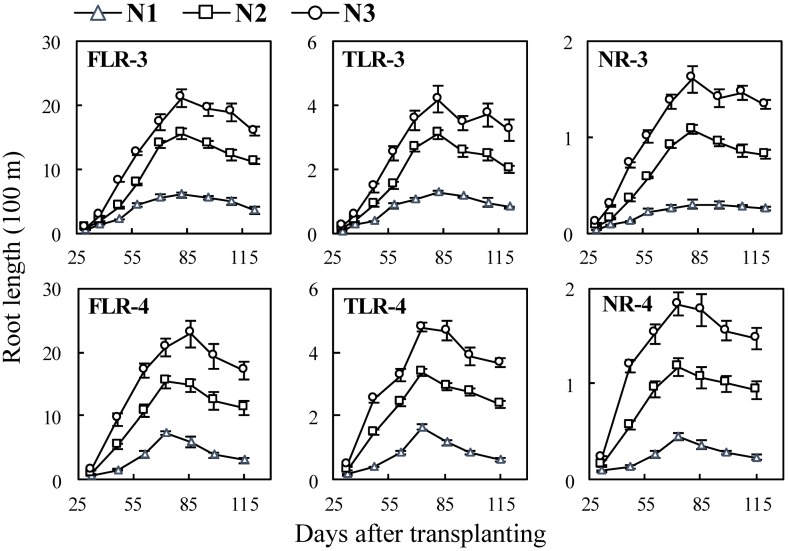
Changes in length of three types of rice roots in N experiments following transplanting. FLR, TLR and NR denote different root types: fine lateral roots, thick lateral roots, and nodal roots, respectively. The numbers following the letters denote Exp.3 and 4, and the cultivar ‘Wuxiangjing 14’ was used. N1, 0 N; N2, 150 kg N ha^−1^; N3, 300 kg N ha^−1^. The values are the means of 3 replicates (± *SE*).

In the N experiments (Fig 6), increasing N rate accelerated the growth and branching rate of roots, which resulted in the length differences of three types of rice roots among different N rates in Exp. 3 and Exp. 4. For example, the average total length of NRs under N3 reached 161.3 m but was only 27.0 m under N1 at the 82 DAT in Exp. 2 (Fig 6 FLR-3).

**Fig 7:**
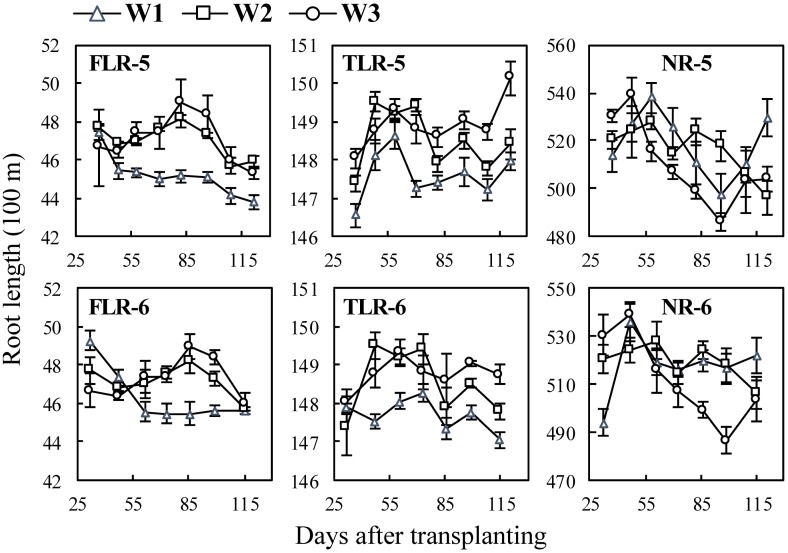
Changes in length of three types of rice roots in water experiment following transplanting. FLR, TLR and NR denote different root types: fine lateral roots, thick lateral roots, and nodal roots, respectively. The numbers following the letters denote Exp.5 and 6, and the cultivar ‘Wuxiangjing 14’ was used. W1, keeping 1–2 cm water layer; W2, wetting irrigation; W3, dry cultivation. The values are the means of 3 replicates (± *SE*).

In the water experiments (Fig 7), with the water treatments, lengths of lateral roots under W2 treatment increased faster than those under W1 and W3 treatments, but the lengths of nodal roots under W2 treatment increased nearly as fast as those of W1. The length of all three types of roots under W3 increased faster at the early stages of the treatment than the later stages, and their peak values were the lowest.

### Dynamics of surface area percentage of three types of roots with growth stages

**Fig 8:**
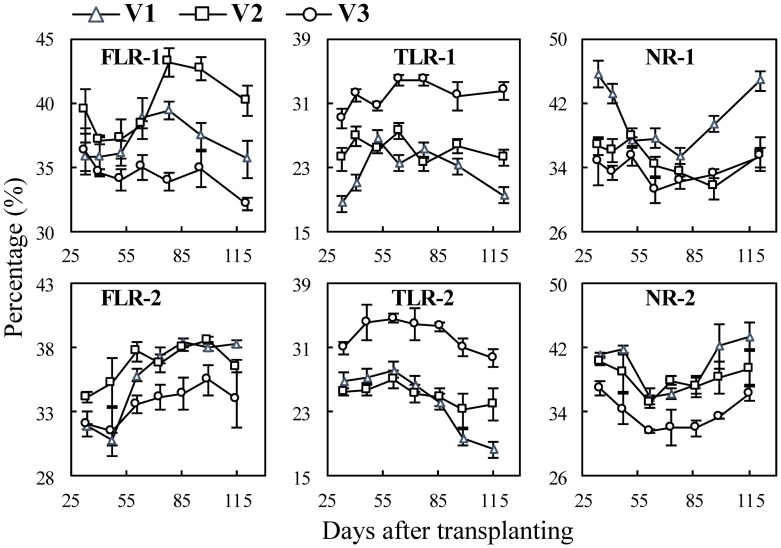
Changes in surface area percentage of three types of rice roots in cultivar experiments following transplanting. FLR, TLR and NR denote: fine lateral root, thick lateral root, and nodal root, and the numbers following the letters denote Exp. 1 and 2, respectively. V1, ‘Nipponbare’; V2, ‘Wuxiangjing 14’; V3, ‘Yangdao 6’. The values are the means of 3 replicates (± *SE*).

**Fig 9:**
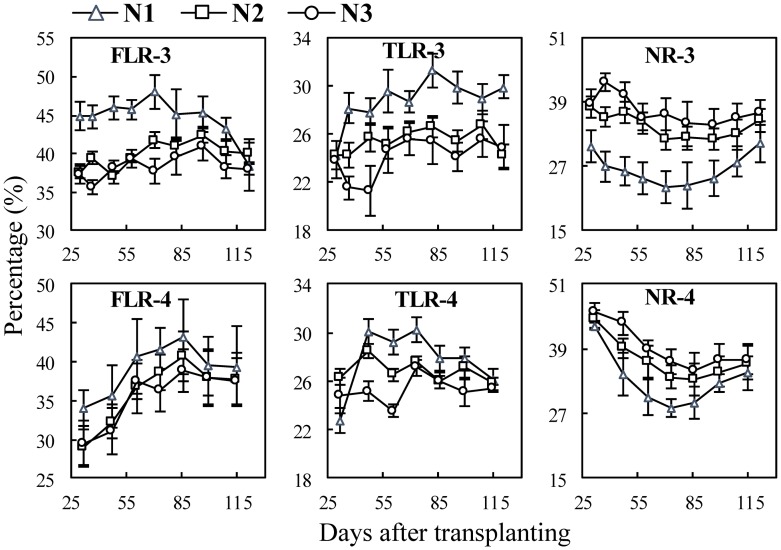
Changes in surface area percentage of three types of rice roots in N experiments following transplanting. FLR, TLR and NR denote different root types: fine lateral roots, thick lateral roots, and nodal roots, respectively. The numbers following the letters denote Exp.3 and 4, and the cultivar ‘Wuxiangjing 14’ was used. N1, 0 N; N2, 150 kg N ha^−1^; N3, 300 kg N ha^−1^. The values are the means of 3 replicates (± *SE*).

**Fig 10:**
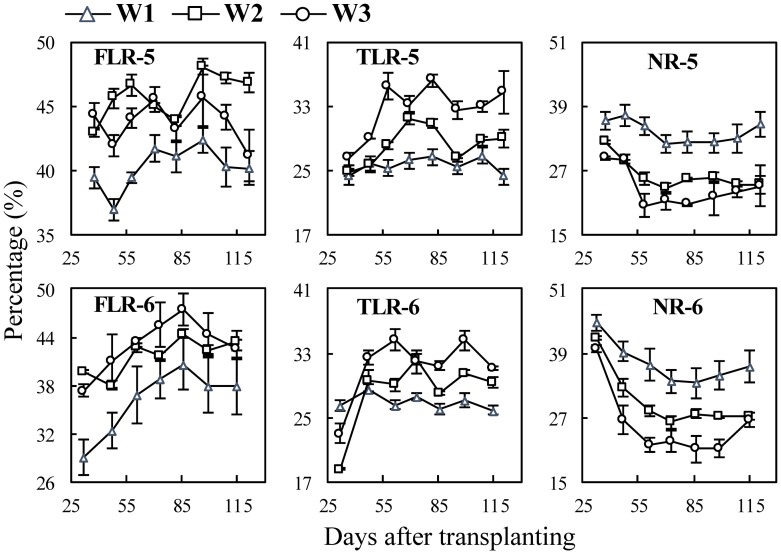
Changes in surface area percentage of three types of rice roots in water experiments following transplanting. FLR, TLR and NR denote different root types: fine lateral roots, thick lateral roots, and nodal roots, respectively. The numbers following the letters denote Exp.5 and 6, and the cultivar ‘Wuxiangjing 14’ was used. W1, keeping 1–2 cm water layer; W2, wetting irrigation; W3, dry cultivation. The values are the means of 3 replicates (± *SE*).

The changing trends of surface area with growth stages were similar with those of root length (see S5 and S6 Files), thereby only the dynamics of their surface area percentages (SAP) in rice plant are presented. In the cultivar experiments (Fig 8), for different cultivars, the SAP of fine lateral roots (SAP_F_) and nodal roots (SAP_N_) of V3 were significantly lower than V1 and V2, but contrary to the SAP of thick lateral roots (SAP_T_). The difference between V1 and V2 were relatively smaller. In the N experiments (Fig 9), SAP_F_ and SAP_T_ decreased and SAP_N_ increased with nitrogen rates. In the water experiments (Fig 10), SAP_F_ and SAP_T_, generally decreased with the increase of soil water content, e.g. W3>W2>W1, except for the SAP_F_ in Exp. 5 which was W3>W1>W2 in several stages. The SAP_N_ were W1>W2>W3, and there was significant difference among the three treatments.

## Discussion

Many studies have analyzed root morphology using the *WinRhizo* image analysis system [35–37]. However, most of these studies focused on physiological or total root morphological characters [25, 33, 38–41]. In contrast, this study managed to differentiate three types of rice roots by means of data classification using an image analysis method under different nitrogen and water conditions.

Although monocot roots like rice do not undergo secondary radial growth, there are differences on diameters and lengths among nodal roots that initiated at different main stem and tiller nodes at different development stages and were affected by environmental changing [9, 32]. Therefore, this study aimed to quantitatively determine these differences among different types of rice roots. Some previous studies showed that total root length, active absorbing area and mean diameter of NRs of Indica rice usually larger than those of Japonica rice, but the information was lacked on lateral roots [42, 43]. In the cultivar experiments, the diameters of the TLRs and NRs of the Indica cultivar ‘Yangdao 6’ were significant larger than the two Japonica cultivars, but there were contrary results on the FLRs (Fig 2). The length and surface area percentage of FLRs and TLRs of V3 significantly higher than those of V1 and V2 (Figs 5 and 7), indicating V3 has better branching ability on lateral roots, especially on the TLRs that could be helpful to further expand soil space based on the growth of NRs. These differences of root morphology among the cultivars showed different nutrient and water uptake patterns. Indica rice usually has a larger root system (such as biomass and length), but it doesn’t induce a significantly higher yield. Thus, the field management measures and breeding improvement should take account the root morphology traits [42–44].

Most of FLRs at early stage emerged on nodal roots, while a great proportion of FLRs at late stages emerged on TLRs which diameters are smaller. Thus, the diameter of FLRs decreased before 48 DATs (Fig 2 FLR-1 and 2, Fig 3 FLR-1 and 2). However, these tendencies were not significant under 0 N treatments and water controlled treatments (W2 and W3), this may because higher order lateral roots were decreased under nitrogen and water deficiency conditions. The similar results also showed in the previous study that lateral roots are easy to wither, particularly under unsuitable conditions such as poor soil and drought conditions [33].

Higher N supply typically generates more tillers and roots on tillers, and also accelerates root growth and branching [45, 46]. This study showed the similar results on root length and surface area. The results also showed that the diameter and surface area percentage of NRs increased with the increase of N rates (Fig 6 NR-3 and 4; Fig 9 NR-3 and 4), indicating that the relative lower root biomass leaded to thinner NRs. However, the diameter of FLRs and TLRs showed no significant differences between N treatments (Fig 3 FLR-3, TLR-3, FLR-4 and TLR-4), while surface area percentage of FLRs and TLRs decreased with the increase of N rates (Fig 9 FLR-3, TLR-3, FLR-4 and TLR-4), this indicated that rice plant tends to grow lateral roots under N stress conditions.

Previous studies showed that appropriate water stress induces the growth of roots [33, 39]. This study also showed the similar results that the root length of wet water treatment was the longest, while that of dry treatment was the shortest (Fig 7) due to too little root biomass accumulated under severe water stress condition. The diameters and surface area percentage of lateral roots under water stress treatments (W2, W3) were higher than those under water layer treatment (W1). This suggested that lateral roots also have the priority to growth under water stress conditions. The previous studies showed that the ratio of root to shoot increased under stress conditions [3, 9, 39, 47, 48]. These conclusions suggested that rice plant tends to increase root uptake area using limiting available assimilates to increase root uptake area under stress conditions.

## Supporting Information

S1 File Root image analysis method. S1 File shows the root image analysis method that used in this study.
(DOCX)

S2 File Data for the figures in S1 File. S2 File shows the data and figures in S1 File.
(XLSX)

S3 File Data for Figs 2–10. S3 File shows the data for Figs 2–10.
(XLSX)

S4 File Raw data for each root image. S4 File shows the raw data for of each root image used for Figs 2–10.
(XLSX)

S5 File Changes in surface area of three types of rice roots. S5 File shows the changes in surface area of three types of rice roots.
(DOCX)

S6 File Data for the surface area of three types of rice roots. S6 File shows the data of the surface area of three types of rice roots.
(XLSX)
