# Supplementary material for: Quantitative Classification of Rice (Oryza sativa L.) Root Length and Diameter Using Image Analysis
Source: PLoS One. 2017 Jan 19;12(1):e0169968. doi: 10.1371/journal.pone.0169968 (PMC5245893; doi:10.1371/journal.pone.0169968)
Supplement: S1 File — shows the root image analysis method that used in this study. (DOCX) [file pone.0169968.s001.docx]

# Root image analysis method

# Method

# Software for root image analysis

Two software programs were used to analyze root images in this study: *WinRhizo* (version 5.0) and *Image–Pro plus* (version 6.0) (Media Cybernetics, Inc., Silver Spring, USA). The *WinRhizo* calculates root length by multiplying pixel number by pixel size and measures average diameter by dividing the projected area of the imaged object by the total length. Root lengths are assigned to predefined diameter classes, thus providing diameter distributions of the total root system. The *Image–Pro plus*’s line and curve measurement tool were used to verify the accuracy of root diameters and lengths obtained with *WinRhizo*. Another software *TechSmith Snagit* 9.0 (TechSmith Co., Ltd, USA) was used to capture and save screen images.

# Image resolution

In order to check the measurement accuracy of root diameters, 20 grayscale images were scanned twice at resolutions of 400 dpi (with the *WinRhizo*) and 4000 dpi (with the *Image–Pro plus*). The 4000 dpi images were used by *Image–Pro plus* to obtain a high measurement accuracy of root diameter, and root diameters measured in the same positions were compared for the two resolutions. An image resolution value of 400 dpi was selected for samples analysed by *WinRhizo* based on acceptable measurement accuracy and reduced scanning time, analysis time, and disk storage requirements.

# Threshold value setting in *WinRhizo*

Setting an appropriate threshold value for root analysis was an important step to ensure that the different diameter classes of rice roots were properly analysed from the grayscale images. Threshold values could be adjusted by 3 methods provided by *WinRhizo*: (1) an automatic threshold, (2) Lagarde’s method and (3) a manual threshold. Root diameter values may be overestimated by using an excessively high grayscale threshold value. However, very fine roots may be missed by using a threshold value that is too low (Regent Instruments, Inc., 2005). For example, Fig.S1 A is the original image obtained from the scanner. Threshold value of 176 was selected by *WinRhizo* using the automatic method, the nodal root (NR) and thick lateral roots (FLRs) were properly analysed, but most of the fine lateral root (TLRs) were missed (Fig.S1 B). When threshold value of 200 was specified manually, the NR and most of TLRs were properly analysed, but only a few FLRs were detected (Fig S1 C). In Fig.S1 D, when the Lagarde’s method was used, most of the lateral roots were detected, but the diameters of NR were overestimated. After a series of comparisons of the accuracy of root diameters with varied threshold values, Lagarde’s method and threshold value of 200 were used to obtain data in the 0-250 μm and 250-2000 μm diameter ranges, respectively.


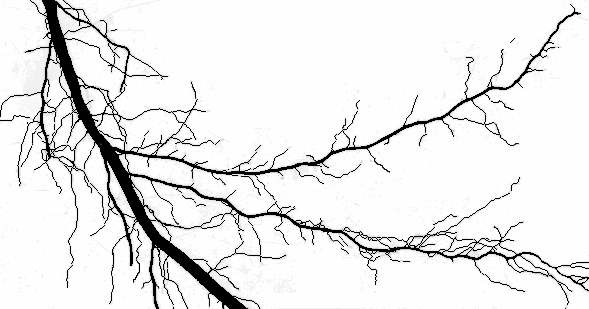

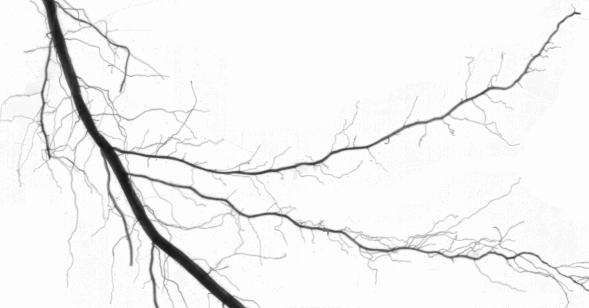

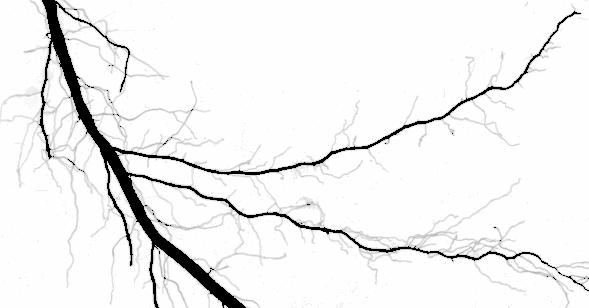

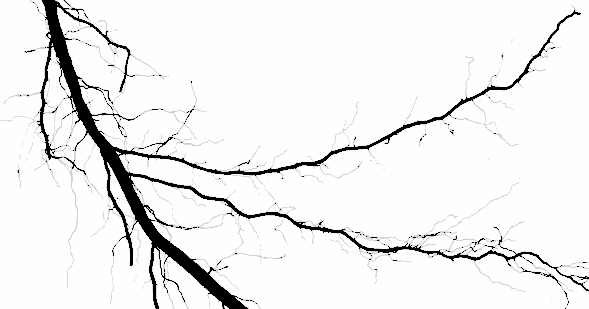


**TLR**

**FLR**

**NR**

**TLR**

**Fig S1. Images of rice roots obtained with different threshold values.** A: Original image B: Image analyzed by *WinRhizo* using the automatic threshold setting method (threshold value = 176). C: Image from manually selected method (threshold value =200). D: Image analyzed by *WinRhizo* using Lagarde’s method. In panels B, C and D, the dark black segments represent roots measured by *WinRhizo*; light grey segments were not measured by *WinRhizo*.

# Determination of diameter range for different types of rice root

Three types of rice roots were evaluated in this study: nodal root (NR), fine lateral roots (FLR) or thick lateral root (TLR). The rice roots have no secondary thickening and the diameters were normally in the order of NR > TLR > FLR [1-4]. Based on root diameter and the location where the root emerged, most root types were easily identified in the images. 400 images (>30% of the total images) per treatment were selected randomly, and the diameters of the three root types in the selected images were measured by *WinRhizo*, with diameter ranges of the roots determined based on the sample distribution. In addition, data for the lengths of the three types of rice root also were [classified](app:ds:classification) according to diameter range. 50 μm was used as the interval of the range, e.g. the diameter range was divided into: 0-50, 50-100, 100-150……and 1950-2000 μm.

# Accuracy of root length measurements

The *Image–Pro plus* was used to validate the accuracy and precision of root length data obtained by the *WinRhizo* program. To measure the lengths of the three types of roots, results were compared with those obtained by *WinRhizo*. 150 root images were selected randomly for each treatment, and the lengths of the nodal root axes in each of the images were measured manually with a curve measurement tool in *Image–Pro plus*. Due to the large quantity of lateral roots, a small area (2.526 cm^2^) of an image was analysed by *WinRhizo*, and this area was captured and saved by using *TechSmith Snagit* 9.0. 50 images were selected for each treatment and each type of lateral root. Lengths of the lateral roots in these small areas were also manually measured by the *Image–Pro plus.*

# Statistics analysis

The images obtained in experiments 1, 3, and 5 were used to determine the root diameter ranges of the three types of root. Images obtained in experiments 2, 4, and 6 were used for the validation of results. The mean diameter of the three types of rice roots were calculated based on their length (L) and surface area (SA) using the following equation:

*$Diameter=\frac{SA}{\pi\times L}$*.

*R^2^* and normalized root mean squared error (*NRMSE*) were used to calculate the fitness between the values obtained by *WinRhizo* and by *Image–Pro plus* [5]. Data were subjected to a one-way ANOVA and significant differences of treatment means were compared by the Fisher's protected least significant difference (LSD) procedure with the SPSS 17.0 (SPSS Inc., 2008).

# Results

# Comparisons of root diameters in 200-350 μm measured by two threshold value setting in *WinRhizo*

The root diameters measured with WR using the Lagarde’s method and threshold value of 200 were compared with IPP in 200-350 μm, and the changes in relative error were showed in Fig S2. The relative error increased with the root diameter when the Lagarde’s method was used, (Fig S2 A). But the relative error decreased with the increasing of the root diameter when the threshold value of 200 was used (Fig S2 B). Thus, these two threshold value settings could be used for different root diameter ranges.

**Fig S2. Changes in relative error (%)**= **of root diameters measured by WR and IPP in 200–350 μm range.** Relative error (%)= (Root diameter measured by IPP - Root diameter measured by WR)/Root diameter measured by IPP. Root diameters were measured with two kinds of images: those of 400 dpi (by WR) and those of 4000 dpi (by IPP). The root diameters measured with WR using Lagarde’s method and threshold value of 200 correspond to figures A and B, respectively.

# Comparisons of root diameters measured by *WinRhizo* and *Image–Pro plus*

The measurement accuracy of root diameter was further evaluated in three ranges: 0-100 μm, 100-250 μm and 250-2000 μm (Fig S3). Diameter values obtained by *WinRhizo* and *Image–Pro plus* were closely related and the fitness increased with increasing root diameter. Diameter measured by *WinRhizo* using Lagarde’s method for 0-100 μm (Fig S3 A) and 100-250 μm (Fig S3 B) roots were compared with the diameters measured by *Image–Pro plus* with *R^2^* values of 0.937 and 0.949, *NRMSEs* of 12.15% and 6.45%, respectively. Diameter values measured by *WinRhizo* with a threshold value of 200 for 250-2000 μm roots were compared with *Image–Pro plus* with a *R^2^* of 0.996 and *NRMSE* of 4.12% (Fig S3).

**Fig S3. Comparison of rice root diameters measured by *WinRhizo* and *Image–Pro plus* for three diameter ranges.** Root diameters were measured with two image resolutions: (1) 400 dpi (by *WinRhizo*) and (2) 4000 dpi (by *Image–Pro plus*). Two kinds of threshold setting were used in *WinRhizo*: Lagarde’s method for 0-100 μm (A) and 100-250 μm (B), and a threshold value of 200 for 250–2000 μm roots (C). ***p* < 0.01.

# Classification of three types of root based on diameter range

The diameters of three types of root were investigated by random sampling based on the *WinRhizo*. Results are shown in Fig. S4. In cultivar experiment (Exp. 1), 63.0-75.3% of FLR diameters were concentrated in the 30-40 μm range, and none was found over 0.11 μm. 48.5-79.3% and 65.3-77.8% of TLR and NR diameters were found in the 100-150 μm and 400-1000 μm ranges, respectively. Cultivar Yangdao 6 (V3) had a greater percentage of TLRs and NRs of large diameters than the other two cultivars in the ranges of 150-200 μm and 1.00-2.00 μm. Nipponbare (V1) and Wuxiangjing 14 (V2) did not differ significantly in diameter distribution (Fig S4 FLR-1, TLR-1, and NR-1).

In N experiment (Exp. 3), there was no significant difference in diameter distribution among the three N treatments (Fig S4 FLR-3, TLR-3, and NR-3), In water experiment (Exp. 5), the lower water supply increased the distribution percentage of FLRs and TLRs within the 50-100 μm and 150-200 μm ranges (Fig S4 FLR-5, TLR-5, and NR-5), but the main distribution ranges of FLRs and TLRs were 0-100 μm and 100-250 μm under the three water regimes. And the main distribution ranges of NRs were small differences among the three treatments.

**Fig S4. Percentage of roots in each diameter class as measured by *WinRhizo*.** FLR, TLR and NR denote different root types: fine lateral roots, thick lateral roots, and nodal roots, respectively. The numbers following the letters denote Exp.1, 3 and 5. Cultivars treatments in Exp.1: V1, ‘Nipponbare’; V2, ‘Wuxiangjing 14’; V3, ‘Yangdao 6’. N treatments in Exp.3: N1, 0 N; N2, 150 kg N ha^−1^; N3, 300 kg N ha^−1^. Water treatments in Exp.5: W1, keeping 1-2 cm water layer; W2, wetting irrigation; W3, dry cultivation. The cultivar ‘Wuxiangjing 14’ was used in N and water experiments.

Based on the above analysis, the diameter ranges of the three types of rice roots were classified (Table S1). More than 99.0% of the sampling diameters of FLRs in three cultivars under different treatments were in the range of 0-100 μm. 98.8 and 96.8% of the diameters of TLRs and NRs of Yangdao 6 (V3) were in the range of 100-300 μm and 300-1950 μm, respectively, while over 91.5 and 96.3% of the diameters of TLRs and NRs of the other two cultivars were found between 100-250 μm and 250-1800 μm. Therefore, under different N and water treatments, 90.0 to 100% of the FLRs, TLRs, and NRs were in the ranges of 0-100 μm, 100-250 μm, and 250-1800 μm, respectively.

**Table S1 Diameter ranges of different types of rice roots**

| **Experiment** | **Treatment** | **Fine lateral root** | | **Thick lateral root** | | **Nodal root** | |
| --- | --- | --- | --- | --- | --- | --- | --- |
|  |  | **Diameter range (μm)** | **Percentage of random sampling (%)** | **Diameter range (μm)** | **Percentage of random sampling (%)** | **Diameter range (μm)** | **Percentage of random sampling (%)** |
| **Exp.1** | **V1** | 0–100 | 99.5 | 100–250 | 93.0 | 250-1800 | 97.8 |
|  | **V2** | 0–100 | 99.3 | 100–250 | 91.5 | 250-1800 | 96.3 |
|  | **V3** | 0–100 | 99.5 | 100–300 | 98.8 | 300-1950 | 96.8 |
|  |  |  |  |  |  |  |  |
| **Exp.3** | **N1** | 0–100 | 99.5 | 100–250 | 90.0 | 250-1800 | 98.5 |
|  | **N2** | 0–100 | 99.8 | 100–250 | 91.0 | 250-1800 | 97.3 |
|  | **N3** | 0–100 | 99.5 | 100–250 | 91.8 | 250-1800 | 95.3 |
|  |  |  |  |  |  |  |  |
| **Exp.5** | **W1** | 0–100 | 100.0 | 100–250 | 91.5 | 250-1800 | 96.8 |
|  | **W2** | 0–100 | 99.0 | 100–250 | 90.3 | 250-1800 | 95.5 |
|  | **W3** | 0–100 | 99.3 | 100–250 | 92.3 | 250-1800 | 96.3 |

# Comparisons of root length measured by *WinRhizo* and *Image–Pro plus*

The lengths of the three types of rice roots were obtained based on the previously described three diameter ranges using independent datasets. Close agreement was found between *WinRhizo* and *Image–Pro plus* measurements (Fig S5). The linear regression line between the two sets of values was close to the 1:1 line, and the *R^2^* values for each root type under each treatment were all in the range of 0.951-0.990. The *NRMSE*s for FLR were less than 10% for all cultivars under all treatments, except for N1 (10.13%) and W3 (11.29%). Most of the *NRMSE*s for TLRs were greater than 10% but less than 12.90%. The *NRMSE*s of NRs were between 8.26 and 10.99%.

**Root diameter range (cm)**

**Root length measured by IP (cm)**

**Fig S5. Comparison of *WinRhizo* and *Image–Pro plus* measurement of root length for different types of roots.** ***p* < 0.01. FLR, TLR and NR denote different root types: fine lateral roots, thick lateral roots, and nodal roots, respectively. The numbers following the letters denote Exp.2, 4 and 6. Cultivars treatments in Exp.2: V1, ‘Nipponbare’; V2, ‘Wuxiangjing 14’; V3, ‘Yangdao 6’. N treatments in Exp. 4: N1, 0 g pot^–1^ N; N2, 1.5 g·pot^–1^ N; N3, 3.0 g pot^–1^ N. Water treatments in Exp. 6: W1, keeping 1-2 cm water layer; W2, wetting irrigation; W3, dry cultivation. The cultivar ‘Wuxiangjing 14’ was used in N and water experiments.

# Discussion

In this study, two methods were evaluated for setting threshold values in *WinRhizo*: the Lagarde’s method was more reliable for measuring lateral roots, and the threshold value of 200 was selected and validated to effectively measure nodal roots. Root diameter values may be overestimated by using an excessively high grayscale threshold value. However, very fine roots may be missed by using a threshold value that is too low (Regent Instruments, Inc., 2005).

Higher image resolution (e.g. 4000 dpi) would allow for better discrimination of root morphological data, but requiring more scanning and analysis time and storage space. problematic for large numbers of samples such as in this study, in which more than 2000 root images were to be analysed representing several experiments, samplings, replications, phenological stages, growing seasons, varieties, nitrogen levels, and water regimes. Considering time, storage, and accuracy requirements, a reasonable image resolution (i.e. 400 dpi) was chosen for an image analysis method. In addition, for this purpose, an appropriate parameter setting (i.e. threshold value) was required for the *WinRhizo* software [6, 7]. Based on the parameter setting for evaluating a large number of root images, the batch process function of *WinRhizo* was used to improve image analysis efficiency.

**References**

1. Xu SX, Xu XB, He YK. Morphology and Anatomy of Rice. Beijing: Agricultural Press; 1984. (in Chinese)

2. Kawada S. Root System of Rice (Translated by Shen TX, Liu ZJ, Peng WY). Beijing: Agriculture Press; 1984. (in Chinese)

3. Morita S, Abe J. Roots and nitrogen in cropping systems of the semi-arid tropics. In: Ito O, Johansen C, Adu-Gyamfi J, Katayama K, Kumar Rao J, Rego T, editors. Development of root system in wheat and rice. Tokyo: Japan International Research Center for Agricultural Sciences; 1996. pp.185-198.

4. Li YH. Morphological and anatomy of gramineous crop. Shanghai: Shanghai Scientific and Technical Publishers; 1979. (in Chinese)

5. Wallach D, Makowski D, Jones JW. Working with dynamic crop models Amsterdam, Boston: Elsevier; 2006.

6. Himmelbauer ML, Loiskandl W, Kastanek F. Estimating length, average diameter and surface area of roots using two different image analyses systems. Plant Soil. 2004; 260: 111-120. doi: 10.1023/B: PLSO.0000030171.28821.55.

7. Bouma TJ, Nielsen KL, Koutstaal B. Sample preparation and scanning protocol for computerised analysis of root length and diameter. Plant Soil. 2000; 218: 185-196. doi: 10.1023/A:1014905104017.
