# Supplementary material for: Quantitative Classification of Rice (Oryza sativa L.) Root Length and Diameter Using Image Analysis
Source: PLoS One. 2017 Jan 19;12(1):e0169968. doi: 10.1371/journal.pone.0169968 (PMC5245893; doi:10.1371/journal.pone.0169968)
Supplement: S5 File — shows the changes in surface area of three types of rice roots. (DOCX) [file pone.0169968.s005.docx]

**Days after transplanting**

**Surface area (cm^2^)**

**Fig S6. Changes in surface area of three types roots in cultivar experiments following transplanting.** FLR, TLR and NR denote: fine lateral root, thick lateral root, and nodal root, and the numbers following the letters denote Exp. 1 and 2, respectively. V1, ‘Nipponbare’; V2, ‘Wuxiangjing 14’; V3, ‘Yangdao 6’. The values are the means of 3 replicates (± *SE*).

**Surface area (cm^2^)**

**Days after transplanting**

**Fig S7. Changes in surface area of three types roots in N experiments following transplanting.** FLR, TLR and NR denote different root types: fine lateral roots, thick lateral roots, and nodal roots, respectively. The numbers following the letters denote Exp.3 and 4, and the cultivar ‘Wuxiangjing 14’ was used. N1, 0 N; N2, 150 kg N ha^−1^; N3, 300 kg N ha^−1^. The values are the means of 3 replicates (± *SE*).

**Days after transplanting**

**Surface area (cm^2^)**

**Fig S8. Changes in surface area of three types roots in water experiments following transplanting.** FLR, TLR and NR denote different root types: fine lateral roots, thick lateral roots, and nodal roots, respectively. The numbers following the letters denote Exp.5 and 6, and the cultivar ‘Wuxiangjing 14’ was used. W1, keeping 1-2 cm water layer; W2, wetting irrigation; W3, dry cultivation. The values are the means of 3 replicates (± *SE*).
